# Mechanical Properties and Cutting Performance of Si_3_N_4_/Sc_2_W_3_O_12_ Composite Ceramic Tools Materials

**DOI:** 10.3390/ma18153440

**Published:** 2025-07-22

**Authors:** Zhiyuan Zhang, Xiaolan Bai, Jingjie Zhang, Mingdong Yi, Guangchun Xiao, Tingting Zhou, Hui Chen, Zhaoqiang Chen, Chonghai Xu

**Affiliations:** 1 School of Mechanical Engineering, Shandong Key Laboratory of CNC Machine Tool Functional Components, Qilu University of Technology (Shandong Academy of Sciences), Jinan 250353, China; m15564460625@163.com (Z.Z.); zjj@qlu.edu.cn (J.Z.); 15053103340@163.com (M.Y.); xgc@qlu.edu.cn (G.X.); zhoutingting506@163.com (T.Z.); chenhui@qlu.edu.cn (H.C.); czq@qlu.edu.cn (Z.C.); xch@qlu.edu.cn (C.X.); 2 Shandong Institute of Mechanical Design and Research, Jinan 250031, China

**Keywords:** Si_3_N_4_/Sc_2_W_3_O_12_ ceramic tool materials, negative thermal expansion, residual stress regulation, spark plasma sintering, cutting performance

## Abstract

To address the poor thermal shock resistance and high brittleness of traditional ceramic tools, a novel Si_3_N_4_/Sc_2_W_3_O_12_ (SNS) composite ceramic material was developed via in situ synthesis using WO_3_ and Sc_2_O_3_ as precursors and consolidated by spark plasma sintering. Sc_2_W_3_O_12_ with negative thermal expansion was introduced to compensate for matrix shrinkage and modulate interfacial stress. The effects of varying Sc_2_W_3_O_12_ content on thermal expansion, residual stress, microstructure, and mechanical properties were systematically investigated. Among the compositions, SNS3 (12 wt.% Sc_2_W_3_O_12_) exhibited the best overall performance: relative density of 98.8 ± 0.2%, flexural strength of 712.4 ± 30 MPa, fracture toughness of 7.5 ± 0.3 MPa·m^1/2^, Vickers hardness of 16.3 ± 0.3 GPa, and an average thermal expansion coefficient of 2.81 × 10^−6^·K^−1^. The formation of a spherical chain-like Sc-W-O phase at the grain boundaries created a “hard core–soft shell” interface that enhanced crack resistance and stress buffering. Cutting tests showed that the SNS3 tool reduced workpiece surface roughness by 32.91% and achieved a cutting distance of 9500 m. These results validate the potential of this novel multiphase ceramic system as a promising candidate for high-performance and thermally stable ceramic cutting tools.

## 1. Introduction

Machining is one of the most fundamental and reliable manufacturing methods, and high-speed cutting technology is the most effective approach to improve machining efficiency [1]. Compared to conventional high-speed steel and cemented carbide tools, ceramic tools exhibit excellent hot hardness, high-temperature stability, superior wear resistance, and strong resistance to chemical reactions. When operating under high-speed cutting conditions, ceramic tools can maintain stable cutting performance, effectively reduce chemical interactions between the tool and workpiece materials, and improve the cutting reliability of the tools [2]. Furthermore, the low friction characteristics and anti-adhesion capabilities of ceramic tools make the machining process more stable. They are especially suitable for processing superhard materials that are difficult for traditional tools, simplifying the processing flow and thereby improving production efficiency [3,4]. In conclusion, as a revolutionary material in the modern high-precision processing field, ceramic tools demonstrate significant performance advantages under extreme processing conditions compared to the traditional high-speed steel and hard alloy systems. They provide more effective solutions for contemporary mechanical processing and have become the preferred tools for advanced manufacturing scenarios, such as aerospace precision components and high-strength alloy turning. Ceramic tools represent a significant advancement in the field of cutting processing and are anticipated to drive the innovation of processing technologies [5,6].

However, due to their inherent brittleness, sensitivity to microcrack defects, and uneven residual tensile stress distribution, multiphase composite ceramic tools often suffer a decline in mechanical stability. As a result, under mechanical impacts or intense thermal fluctuations, especially during high-speed dry cutting, they are vulnerable to structural degradation, which negatively affects cutting reliability and overall performance.

Numerous studies have demonstrated that the cutting reliability of ceramic tools, which is often evaluated in terms of tool life under defined cutting conditions, is fundamentally governed by the microstructure and physical properties of the tool materials. Yin [7] analyzed the failure mechanisms of Al_2_O_3_/TiC micro-nanocomposite ceramic tools, revealing that the fracture of the cutting edge and the spalling of the surface layer were attributed to the initiation and propagation of microcracks, which were induced by the tensile stress gradient within the material. Ma et al. [8] demonstrated that TiC whisker-reinforced Si_3_N_4_ ceramic tools exhibit a distinctive characteristic of full layer spillage wear on the rear face, which is markedly different from the crater-like damage observed in SiAlON tools. This dissimilarity can be attributed to the interfacial stress concentration induced by whisker reinforcement, thereby validating that the mechanical properties of ceramic tools play a decisive role in determining their cutting performance. Cui et al. [9,10], through comparative experiments and the application of microscopic damage theory, revealed that fluctuations in material properties, such as hardness and fracture toughness, induced by microstructures like grain size and phase distribution, are the primary factors contributing to reduced tool life. Xing et al. [11], through the synergistic effects of surface texture design and MoS_2_ solid lubricant modification, demonstrated that Al_2_O_3_/TiC ceramic tools can effectively reduce cutting forces and enhance chip morphology during the dry cutting of hardened steel, confirming the critical role of optimizing the mechanical properties and microstructure in improving cutting reliability. Xu [12], based on experimental data obtained from the dry cutting of hardened T10A steel using Si_3_N_4_-based ceramic tools and considering the stochastic distribution characteristics of material properties, developed a wear life prediction model to enable the quantitative assessment of service performance. Liu et al. [13] constructed a thermomechanical-coupled reliability evaluation framework and elucidated the evolution of the flexural strength of carbonitride titanium carbonitride-based tools with temperature, further confirming the critical role of mechanical property variations in determining cutting reliability.

To address the inherent brittleness and microstructural flaws of ceramic cutting tools, recent studies have focused on incorporating reinforcing phases with superior mechanical properties and on refining the microstructure of the matrix. Xing et al. [14] confirmed that the synergistic modification of laser nano-texturing and WS_2_/Zr composite soft coatings led to a substantial enhancement in the performance of Al_2_O_3_/TiC ceramic tools during the dry cutting of hardened steel. Grigoriev et al. [15] optimized the microstructure and interfacial bonding properties of a carbide matrix, ceramic surface layer, and nano-multilayer coating, thereby effectively managing the distribution of thermomechanical stresses and suppressing brittle fracture behavior, which significantly improved the wear resistance and service life of the cutting tool. Gevorkyan et al. [16] optimized the performance of tool materials by reinforcing an Al_2_O_3_ matrix with nano-SiC, achieving significantly superior cutting performance compared to Al_2_O_3_-based composite ceramic tools reinforced with TiC, TiN, and ZrO_2_. Liu et al. [17] synthesized TiC whiskers as a toughening phase via carbothermal reduction and successfully developed a novel TiB_2_-based ceramic tool material. Their research demonstrated that optimizing the content of TiC whiskers and sintering process parameters can effectively refine the grain structure and reduce microscopic defects, ultimately leading to enhanced strength, toughness, and overall cutting performance.

It is widely recognized that the formation of residual stress within composite ceramic tools is closely associated with the thermodynamic interactions among different phases. During the cooling process after sintering, thermal expansion mismatch between the base matrix and reinforcing components causes inconsistent shrinkage behavior. Due to interfacial bonding constraints, these distinct phases cannot deform independently, resulting in the accumulation of localized stress near grain boundaries and adjacent regions of the matrix [18]. When the interfacial bonding strength is insufficient to balance the thermal strain, the release of elastic strain energy between heterogeneous phases induces localized tensile stress concentrations at the microscale. This non-uniform stress distribution is particularly hazardous in brittle ceramic systems. The combined effect of residual tensile stress and the inherently low fracture toughness of ceramic materials can readily initiate and propagate grain boundary microcracks. Furthermore, microstructural imperfections can markedly impair the integrated mechanical behavior of the material. Specifically, the dynamic superposition of residual tensile stress fields and external processing loads exacerbates the damage risk at the cutting edge of the tool, leading to unexpected failures under complex operational conditions [19].

To mitigate residual stress fields arising from thermal expansion mismatch between the matrix and reinforcement phases in composite ceramic tool materials, researchers have focused on optimizing stress distribution via layered structural designs. Du et al. [20] employed a gradient lamination process to construct an interfacial buffer layer, improving the mechanical and cutting performance of composite ceramic tools. Cui et al. [21] developed a multilayer composite structure of Al_2_O_3_/TiB_2_/graphene in ceramic cutting tools to create a gradient distribution of residual compressive stress. This structural strategy effectively adjusted the internal stress levels, thereby enhancing crack resistance and wear performance, which, in turn, contributed to improved cutting efficiency. Lugovy et al. [22] systematically investigated the influence mechanism of residual stress on fracture behavior by fabricating Si_3_N_4_/TiN laminated composite ceramics. Their findings revealed that tensile stress layers can induce unstable crack propagation, whereas compressive stress layers introduced through a rational laminated structure design can effectively suppress crack propagation. Such regulation of the stress gradient has been shown to significantly improve the apparent fracture toughness of the composite material.

Inspired by the successful applications of low thermal expansion ceramics in fields such as microelectronic devices, optical systems, and the aerospace industry [23,24,25,26], researchers have incorporated thermal expansion behavior into the design of composite ceramics as a regulatory approach. By adding negative thermal expansion phases, they effectively reduce residual tensile stresses caused by thermal expansion mismatch. Sun [27] developed a near-zero expansion composite system based on ZrW_2_O_8_ using in situ synthesis technology, which effectively reduced thermal stresses under thermal gradient loading conditions. Pelletant et al. [28] successfully decreased the overall thermal expansion coefficient of ZrO_2_/β-eucryptite and Al_2_O_3_/β-eucryptite composite ceramics by regulating the negative thermal expansion value and phase content and enhanced the flexural strength by utilizing residual compressive stress. Xu [29] substantially improved the flexural strength and thermal shock resistance of SiC-based composites by incorporating negative thermally expanding cordierite, achieving a uniform microstructure and a reduced thermal expansion coefficient. Chen et al. [30] incorporated carbon fibers as a negative thermal expansion phase into SiC porous ceramics. Owing to the relatively high residual compressive stress field distributed within the material, both the strength and thermal shock resistance of the material were significantly improved. Clearly, these studies have demonstrated that introducing a phase with negative thermal expansion into a matrix exhibiting positive thermal expansion can significantly alleviate residual thermal stress in composite ceramics, thereby improving their mechanical strength and resistance to thermal shock. Consequently, incorporating negative thermal expansion (NTE) ceramics as an additive phase into Si_3_N_4_- and Al_2_O_3_-based ceramic cutting tool materials can alter the stress state by adjusting the content of the NTE phase.

To date, there have been relatively few research reports on the incorporation of negative thermal expansion (NTE) phases as reinforcing additives into ceramic cutting tool materials. In particular, the Si_3_N_4_/Sc_2_W_3_O_12_ composite system has not been explored, despite the promising NTE properties of Sc_2_W_3_O_12_ and its potential to effectively regulate residual stresses in ceramic tools. Considering this gap, the present study focuses on the in situ synthesis of Sc_2_W_3_O_12_ within a Si_3_N_4_ matrix, aiming to develop a novel composite ceramic cutting tool material. This work systematically investigates the influence of Sc_2_W_3_O_12_ content on the thermal expansion behavior, residual stress distribution, microstructure evolution, mechanical performance, and cutting reliability of the composite. Furthermore, the toughening and strengthening mechanisms, as well as the dominant wear failure modes of the Si_3_N_4_/Sc_2_W_3_O_12_ composites, are comprehensively analyzed, providing insights for the design of advanced ceramic tool materials with enhanced reliability.

Sc_2_W_3_O_12_ is a structurally stable anisotropic negative thermal expansion (NTE) material, exhibiting a minimum thermal expansion coefficient of −11 × 10^−6^·K^−1^ and demonstrating stable NTE behavior over the temperature range of −263 °C to 1200 °C [31,32,33,34,35]. Currently, commercially available Sc_2_W_3_O_12_ powders fail to satisfy the requirements for use as an additive phase in ceramic tools with respect to both purity and particle size. In this study, an in situ reaction synthesis technique was employed to introduce the precursors Sc_2_O_3_ and WO_3_ into the matrix material, which not only prevented the formation of undesired secondary phases but also ensured a relatively high interfacial bonding strength between the matrix and the NTE additive phase. Additionally, the pretreatment process for the NTE additive phase was eliminated, thereby simplifying the overall preparation process. The chemical reaction equation for the in situ synthesis of Sc_2_W_3_O_12_ is presented as follows:

(1) $${\mathrm{Sc}}_{2}O_{3}{+3\mathrm{WO}}_{3}\to {\mathrm{Sc}}_{2}W_{3}O_{12}$$

Considering the thermal expansion mismatch and chemical compatibility between the negative thermal expansion (NTE) phase Sc_2_W_3_O_12_ and the ceramic matrix, Si_3_N_4_ was selected as the matrix material for the cutting tool due to its relatively low coefficient of thermal expansion (approximately 2.4–4.0 × 10^−6^·K^−1^ in the temperature range of room temperature to 1400 °C), high strength and fracture toughness, excellent wear resistance, and superior thermal and mechanical shock resistance. Al_2_O_3_ and Y_2_O_3_ were used as sintering additives, and spark plasma sintering (SPS) technology was employed. Sc_2_W_3_O_12_ was in situ synthesized within the Si_3_N_4_ matrix, thereby fabricating a Si_3_N_4_/Sc_2_W_3_O_12_ composite ceramic cutting tool system. The research elucidated the mechanism by which the negative thermal expansion behavior of Sc_2_W_3_O_12_ influences residual stress distribution in composite ceramic tool materials. Furthermore, this study thoroughly investigated how varying the Sc_2_W_3_O_12_ content affects the composites’ thermal expansion behavior, internal stress state, mechanical properties, microstructure evolution, and cutting performance. The toughening and strengthening mechanisms, as well as the dominant wear failure modes of the Si_3_N_4_/Sc_2_W_3_O_12_ system, were also systematically analyzed.

## 2. Materials and Methods

### 2.1. Materials and Preparation

The raw powders used in this study included α-phase Si_3_N_4_ (with a mass fraction exceeding 92%), α-Al_2_O_3_, WO_3_, Sc_2_O_3_, and Y_2_O_3_, all of which were supplied by Shanghai Chao Wei Nano Technology Co., Ltd. (Shanghai, China). The key physical parameters of these raw materials are summarized in Table 1.

**Figure 1:**
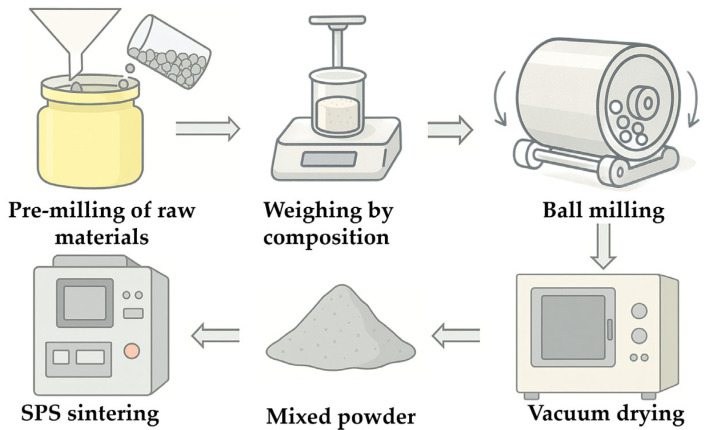
Fabrication flow chart of the composite ceramic.

The primary procedures involved in the preparation process are schematically shown in Figure 1. Before mixing the composite powders, each individual phase powder was subjected to 48 h of pre-ball milling to eliminate uneven particle size distribution and particle agglomeration. During the mixing stage, the powders were weighed according to predetermined component ratios (as shown in Table 2 and Table 3 for the mass compositions of different composites). The accurately weighed powders were then ultrasonically stirred with anhydrous ethanol for 30 min to form a uniformly dispersed suspension. Subsequently, the composite slurry was placed in a ball mill using alumina grinding balls at a powder-to-ball mass ratio of 1:5 and subjected to ball milling for 48 h. The slurry was then dried in a vacuum drying oven (Model ZK-82A, Shanghai, China) at 120 °C, followed by sieving through a 200-mesh standard sieve to obtain the composite powder. The composite powder was finally loaded into a cylindrical graphite mold with an inner diameter of 30 mm and underwent a pre-pressing step lasting 30 min under an axial pressure of 5 MPa. Subsequently, densification and sintering were performed using a SPS-625HF (Fuji Denpa Kogyo Co., Ltd., Tokyo, Japan) plasma-activated sintering apparatus.

Based on the results of residual stress simulation and experimental synergy, composite ceramic tool samples were ultimately designed with Sc_2_W_3_O_12_ contents of 0 wt.%, 8 wt.%, 10 wt.%, 12 wt.%, and 16 wt.% for systematic investigation. According to Equation (1), the molar ratio of Sc_2_O_3_ to WO_3_ is 1:3. Based on this stoichiometric ratio, Sc_2_W_3_O_12_ was in situ synthesized in the composite ceramic materials SNS1, SNS2, SNS3, and SNS4. The corresponding mass fractions of the precursors Sc_2_O_3_ and WO_3_ are listed in Table 2. Five different composition ratios were tested. The details are shown in Table 3.

### 2.2. Material Characterization

The sintered specimens were initially processed into rectangular bars (3.5 mm × 4 mm × 30 mm) by cutting with an inner-circular cutting machine (Model J5060C-1, Shanghai Huisheng Special Radio Technology Co., Ltd., Shanghai, China) and subsequently ground using a precision lapping machine (UNIPOL-1502, Shenyang Kejing Automation Equipment Co., Ltd., Shenyang, China). The edges of each bar were chamfered to approximately 0.1 mm × 0.1 mm and further refined to eliminate surface damage. Final polishing was conducted using diamond spray to achieve a smooth finish. This grinding and polishing sequence helped introduce compressive stresses on the surface [36] and minimized the impact of surface flaws on the mechanical performance. The flexural strength was assessed through a three-point bending test (Model AGS-X5KN, Shimadzu Corporation , Kyoto, Japan) at a span of 20 mm and a loading speed of 0.5 mm/min. Vickers hardness was determined on the polished samples using a Vickers indenter (Model HVS-50, Shandong Shancai Testing Instrument Co., Ltd., Jinan, China) under a 196-N load with a 15-s dwell time. Fracture toughness was evaluated using the Vickers indentation method [37]. Each mechanical parameter was tested using at least six specimens. Only specimens with a relative density exceeding 98% were used for mechanical property evaluation to minimize errors. The density was determined via the Archimedes method using a solid density tester (Model Quarrz AU-200ME, Hangzhou Jinmai Instruments Co., Ltd., Hangzhou, China) and distilled water as the immersion medium, with three replicates for each group. X-ray diffraction (XRD) analysis was performed using a Rigaku Smart Lab SE (Rigaku Corporation, Tokyo, Japan) (Cu Kα radiation) to identify the crystalline phases. The content of Sc_2_W_3_O_12_ was estimated based on the intensity ratios of Sc_2_O_3_ and WO_3_ peaks in the diffraction patterns. The microstructure and elemental composition were observed via a scanning electron microscope integrated with an energy-dispersive spectrometer (Phenom Prox, Phenom-World BV, Eindhoven, The Netherlands); prior to SEM observation, gold sputtering was applied to enhance the imaging clarity. Thermal expansion behavior was tested with a high-temperature dilatometer (DIL402CLASSIC, NETZSCH, Selb, Germany), while surface residual stress was measured using the blind hole technique with a stress detector (Model LM-12, Jinan, China). At least three specimens were analyzed for both thermal expansion and residual stress measurements.

### 2.3. Design of Sintering Processes

**Figure 2:**
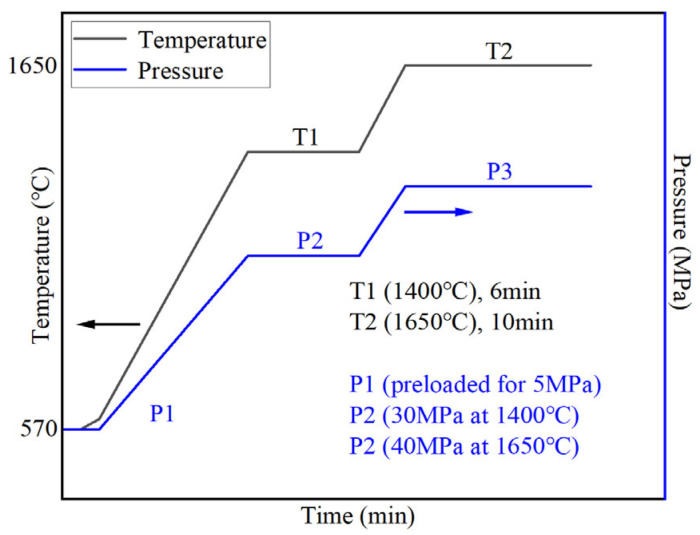
Flowchart of the dual-step heating and pressure protocol during sintering.

According to the experimental investigation of the research group, a partial phase transformation of α-Si_3_N_4_ into β-Si_3_N_4_ occurred using spark plasma sintering Si_3_N_4_/Sc_2_W_3_O_12_ composite ceramic tool materials with Al_2_O_3_/Y_2_O_3_ as a sintering additive in a vacuum atmosphere. Among them, Sc_2_W_3_O_12_ was in situ synthesized in the Si_3_N_4_ matrix through the reaction of the precursors Sc_2_O_3_ and WO_3_ within the temperature range of 1360–1400 °C. Meanwhile, in order to take into account the phase transformation of the silicon nitride matrix and achieve high density, a homogeneous microstructure, and superior mechanical properties, a reasonable design of the sintering process is feasible. To achieve excellent mechanical properties, it is essential to design a reasonable sintering process. Accordingly, the green body was initially preloaded with a pressure of 5 MPa (P1). After evacuating the sintering furnace to a vacuum level of 2.5 × 10^−2^ Pa, a two-step heating and pressure schedule was implemented, as illustrated in Figure 2. Due to the lower display limit of the infrared pyrometer (570 °C), only the portion of the sintering curve above this temperature is illustrated. Before the formal sintering process, a preheating step was conducted up to 570 °C; after which, the temperature was rapidly raised to 600 °C within 1 min. The first step (T1) involved heating from 600 °C to 1400 °C at a rate of 100 °C/min, followed by a 6-min hold at 1400 °C under a sintering pressure of 30 MPa (P2), to facilitate the phase transformation of Si_3_N_4_ and the formation of Sc_2_W_3_O_12_. The second step (T2) continued heating at 100 °C/min up to the final sintering temperature of 1650 °C, maintaining this temperature for 10 min. During this period, the pressure was progressively increased from 30 MPa at 1400 °C to 40 MPa (P3) at 1650 °C, which was then held until the end of the dwell time to promote densification. Finally, the furnace cooled naturally to room temperature. The entire process was conducted in a dual-power spark plasma sintering furnace (SPS-625HF, Fuji Denpa Kogyo Co., Ltd., Tokyo, Japan).

### 2.4. Cutting Test

The composite ceramic tool material SNS3 was selected for single-factor orthogonal cutting tests, with a fixed cutting feed rate of *f* = 0.101 mm/rev. The cutting performance of the tool was investigated under various cutting speeds (*v_c_* = 100 m/min, 150 m/min, 200 m/min, and 250 m/min) and cutting depths (*a_p_* = 0.05 mm, 0.1 mm, 0.15 mm, and 0.2 mm). Based on the results of the orthogonal experiments, the optimal cutting parameters matched to the tool were determined. Subsequently, under these optimal cutting conditions, the cutting performance and tool wear mechanisms of two types of tools, SNS0 and SNS3, were investigated.

**Figure 3:**
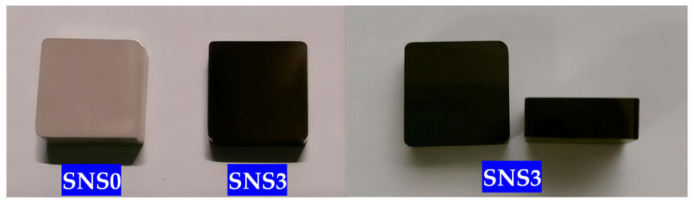
Photographic image of the prepared ceramic inserts SNS0 and SNS3.

In this experiment, the dullness criterion for the durability test of ceramic turning tools as recommended by the international standard ISO was employed. The wear volume VB on the flank face of the tool, set at 0.3 mm, served as the criterion for determining tool failure. The dimensions of the prepared inserts were 12.7 mm × 12.7 mm × 4.7 mm. Figure 3 presents a photographic image of the prepared inserts. The workpiece material was AISI 1045 steel, and the cutting experiments were conducted on a horizontal lathe (CDE6140A, Dalian Machine Tool Group Corporation, Dalian, China) under continuous dry cutting conditions. The geometrical parameters of the cutting tools are listed in Table 4.

**Figure 4:**
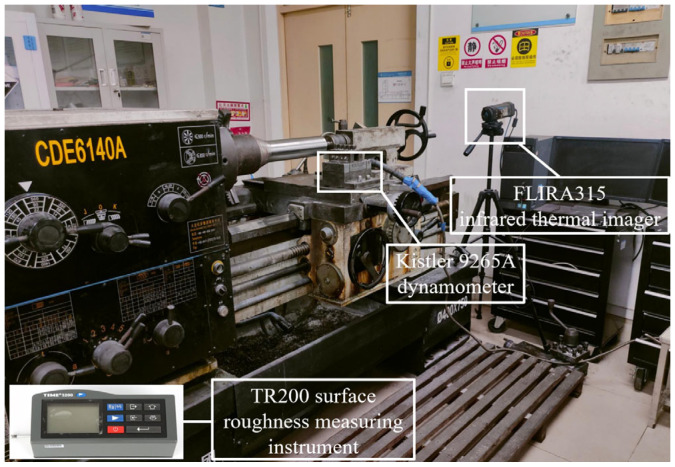
Cutting test scene.

The cutting test scene is presented in Figure 4. Cutting force data were collected using a Kistler dynamometer (model 9265A, Kistler Instrumente AG, Winterthur, Switzerland). Cutting temperature was recorded with an infrared thermal imaging device (FLIRA315-BM, Infratec, Dresden, Germany). A portable device for surface roughness evaluation (TR200, Shanghai Chenyang Instrument Co., Ltd., Shanghai, China) was used to assess the surface finish of the machined samples. The wear on the rake and flank faces was measured using a 3D digital microscope (VHX-5000, Keyence, Osaka, Japan). Additionally, a scanning electron microscope (SEM) integrated with an energy-dispersive spectrometer (EDS) (Phenom Prox, Phenom-World BV, Eindhoven, The Netherlands) was used to observe the microscopic wear morphology on the rake and flank faces of the cutting tools, analyze the chemical composition of the wear regions, and investigate the wear mechanisms of the tools.

## 3. Results and Discussion

### 3.1. Mechanical Properties

**Figure 5:**
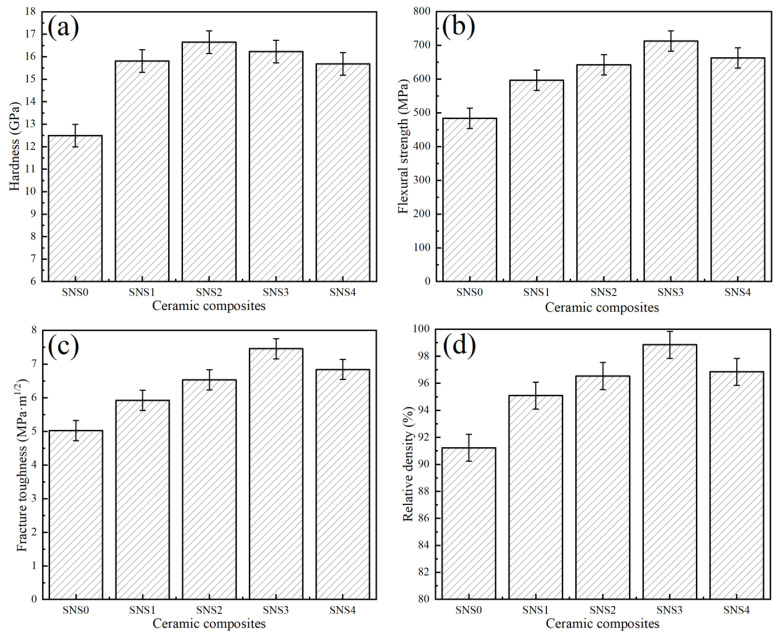
Summary of the mechanical properties and relative densities for five composite samples sintered at 1650 °C with an applied pressure of 40 MPa for 10 min: (**a**) hardness, (**b**) flexural strength, (**c**) fracture toughness, and (**d**) relative density.

Figure 5 illustrates the Vickers hardness, flexural strength, fracture toughness, and relative density of the five Si_3_N_4_/Sc_2_W_3_O_12_ composites sintered at 1650 °C under a pressure of 40 MPa for 10 min. As the Sc_2_W_3_O_12_ content increases, both the mechanical properties and relative density initially improve and then decline. Among them, composite SNS3 containing 12 wt.% Sc_2_W_3_O_12_ exhibits the best overall mechanical performance and highest relative density, with corresponding values of 712.4 ± 30 MPa for flexural strength, 7.5 ± 0.3 MPa·m^1/2^ for fracture toughness, 16.2 ± 0.3 GPa for Vickers hardness, and 98.8 ± 0.2% for relative density.

### 3.2. Microstructure Characterization

**Figure 6:**
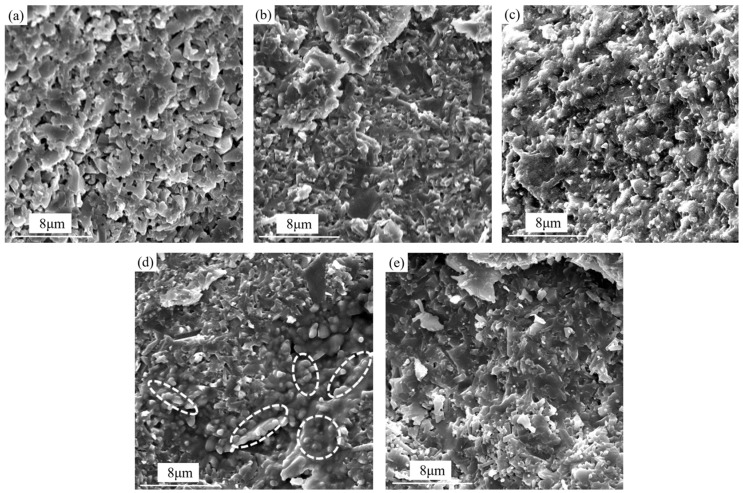
Fracture surface morphologies of the five composite specimens after sintering at 1650 °C under 40 MPa for 10 min (SEM images): (**a**) SNS0, (**b**) SNS1, (**c**) SNS2, (**d**) SNS3, and (**e**) SNS4.

Figure 6 shows the fracture morphologies of the composite ceramic tool materials with different Sc_2_W_3_O_12_ contents. As shown in Figure 6a, the microstructure of the SNS0 material without the Sc_2_W_3_O_12_ addition exhibits a porous morphology, with significant intergranular gaps and irregular pores present between grains. This is attributed to the absence of the negative thermal expansion compensation effect provided by Sc_2_W_3_O_12_. During the cooling process, residual tensile stress is generated between the matrix grains due to contraction, which promotes the formation of interfacial gaps and weakly bonded regions at the grain boundaries. Such structural defects result in the concentration of residual tensile stress, thereby compromising the mechanical properties of SNS0, which correlates with the low hardness, flexural strength, and fracture toughness of the SNS0 material, as depicted in Figure 5. This microstructural characteristic is closely related to the lower hardness, flexural strength, and fracture toughness exhibited by the material shown in Figure 5. These structural defects contribute to the concentration of residual tensile stress, which is directly associated with the relatively low hardness, flexural strength, and fracture toughness observed in Figure 5. It is evident from Figure 6b,c that the addition of 8–10 wt.% Sc_2_W_3_O_12_ results in the Sc-W-O liquid phase filling the pores, thereby facilitating particle rearrangement and significantly reducing porosity. The primary densification effect enables the hardness (as depicted in Figure 5a) to reach a peak value of 16.7 ± 0.3 GPa at 10 wt.%. As shown in Figure 6d, when the Sc_2_W_3_O_12_ content is increased to 12 wt.%, the porosity further decreases. The SC-W-O phase forms a spherical chain network structure at the matrix grain boundaries (indicated by the dotted elliptical box in Figure 6d), with the particle surface becoming smooth and exhibiting no distinct or obvious sharp edges.

As Sc_2_W_3_O_12_ exhibits a negative thermal expansion behavior, it offsets the shrinkage of the matrix during the cooling stage, thereby leading to the formation of a residual compressive stress field with a gradient distribution at the interface between the particles and the matrix. This compressive stress field, on the one hand, suppresses the formation of interfacial defects and minimizes the initiation of pores and microcracks. On the other hand, the residual compressive stress field compels cracks to bypass the spherical particles and undergo path deflection during propagation. As a result, a significant amount of energy is consumed during crack propagation, thereby enhancing the fracture toughness of the material (as illustrated in Figure 5c). In addition, the uniform distribution of Sc_2_W_3_O_12_ particles shown in Figure 6c prevents the formation of localized stress gradients. The curvature characteristics of the spherical morphology further mitigate stress concentration at the grain boundaries, thus enhancing the interfacial bonding strength. Consequently, SNS3 achieves optimal comprehensive mechanical properties, as illustrated in Figure 5. However, when combining the analyses presented in Figure 5 and Figure 6e, it becomes evident that an excessive amount of Sc_2_W_3_O_12_ induces agglomeration of the SC-W-O phase at the grain boundaries, forming a disordered structure. This disrupts the originally uniform compressive stress field, leading to a decline in mechanical properties.

### 3.3. Coefficient of Thermal Expansion and Residual Stress

**Figure 7:**
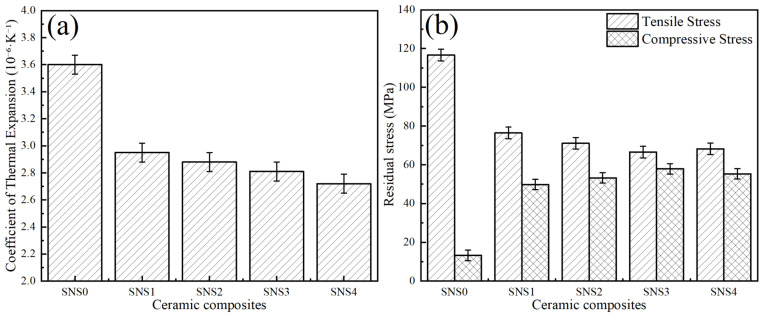
Coefficient of thermal expansion and residual stress of the five composites sintered at 1650 °C under 40 MPa for 10 min: (**a**) coefficient of thermal expansion and (**b**) residual stress.

Since the thermal expansion coefficient of the composite ceramic tool material exhibits a linear combination of the thermal expansion coefficients of each phase with varying contents, the thermal expansion behavior of the composite can be tailored through the controlled variation in the proportions of its individual phases. Moreover, the residual thermal stress within the tool material is influenced by the thermal expansion coefficients of each phase. Consequently, the residual stress in the ceramic tool material can be optimized via an active regulation mechanism of positive and negative thermal expansion matching. Figure 7 illustrates the influence laws of varying Sc_2_W_3_O_12_ contents on the coefficient of thermal expansion (CTE) and residual stresses within the composite ceramic materials. As shown in Figure 7a, as the Sc_2_W_3_O_12_ content increases from 0 wt.% to 16 wt.%, the coefficient of thermal expansion (CTE) of the material decreases continuously from 3.6 × 10^−6^·K^−1^ to 2.72 × 10^−6^·K^−1^. This reduction is attributed to the negative thermal expansion characteristics of the dispersed Sc_2_W_3_O_12_ grains in the matrix, which effectively compensate for the positive thermal expansion of the Si_3_N_4_ matrix. According to the classical theory of composite materials, the effective thermal expansion coefficient of a two-phase ceramic composite can be estimated using the following rule of mixtures model:

(2) $$\partial_{c}=-\frac{\partial_{1}V_{1}E_{1}+\partial_{2}V_{2}E_{2}}{V_{1}E_{1}+V_{2}E_{2}}$$

where *α*, *V*, and *E* represent the thermal expansion coefficient, volume fraction, and elastic modulus of each constituent phase, respectively. This theoretical model suggests that, by increasing the proportion of the negative thermal expansion phase, the overall CTE of the composite can be effectively reduced, thereby mitigating the thermal expansion mismatch stress between the matrix and the reinforcement.

As shown in Figure 7b, with the increasing Sc_2_W_3_O_12_ content, the residual tensile stress decreases from 116.7 ± 14.4 MPa to 66.5 ± 8.7 MPa, while the residual compressive stress increases from 13.3 ± 3.6 MPa to 57.9 ± 4.5 MPa. When the content of Sc_2_W_3_O_12_ was added to 12 wt.%, the residual stress distribution of SNS3 reached its optimal state. Specifically, the tensile and compressive stresses were measured at 66.5 ± 8.7 MPa and 57.9 ± 4.5 MPa, respectively. Compared to SNS0 without Sc_2_W_3_O_12_, the tensile stress of SNS3 decreased by approximately 43%, while the compressive stress of SNS3 increased by approximately 335%. This stress gradient state can effectively passivate crack tips and inhibit crack propagation, which corresponds with the superior comprehensive mechanical properties exhibited by SNS3 in Figure 5. As illustrated in Figure 7, when the content of Sc_2_W_3_O_12_ increases to 16 wt.%, the residual compressive stress exhibits a slight decrease while the tensile stress demonstrates a minor rebound. This increase in the tensile stress state can be attributed to the observed agglomeration of the Sc-W-O phase clusters (as seen in Figure 6e), which suggests that excessive addition compromises the uniformity of the stress field distribution.

In addition, during the cooling process of the SNS3 tool material preparation, the negative thermal expansion property of Sc_2_W_3_O_12_ in the Si_3_N_4_ matrix induces expansion of the matrix, thereby subjecting it to compressive stress. Meanwhile, the differing thermal expansion coefficients of the phases within the composite material result in varying contraction rates, which generate residual tensile and compressive stresses within the matrix. Due to the higher internal compressive stress level in the material compared to the tensile stress level, the stress at the grain boundaries of the matrix is reconfigured into a stress field that is predominantly characterized by compressive stress.

### 3.4. Phase Composition

**Figure 8:**
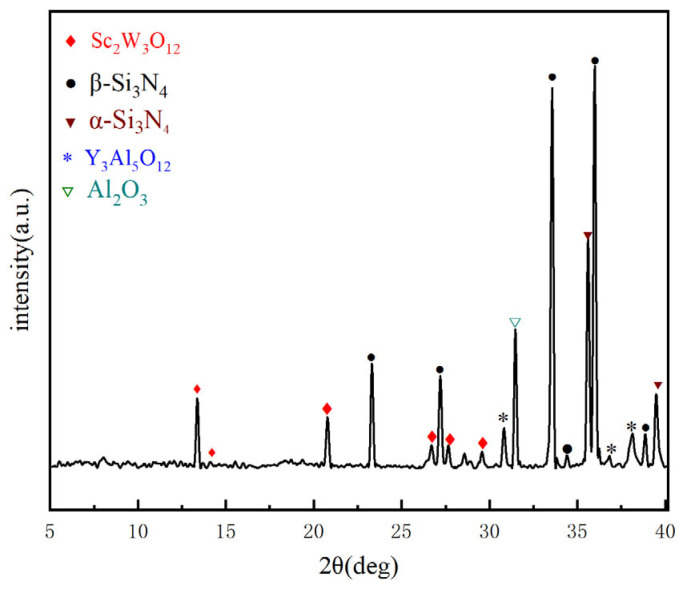
XRD patterns of SNS3.

Figure 8 illustrates the XRD pattern of the composite ceramic material SNS3, which exhibits the optimal comprehensive mechanical properties. As depicted in the figure, SNS3 is composed of Sc_2_W_3_O_12_, β-Si_3_N_4_, α-Si_3_N_4_, Y_3_Al_5_O_12_, and Al_2_O_3_, with no residual Sc_2_O_3_ or WO_3_ detected, indicating that the reaction between Sc_2_O_3_ and WO_3_ was complete. It can also be observed from the figure that the characteristic peak group of the matrix phase β-Si_3_N_4_ (PDF# 33-1160) dominates in intensity, and the characteristic peaks of Sc_2_W_3_O_12_ (PDF# 19-1127) are distinctly identifiable. The sintering additives Y_2_O_3_ and Al_2_O_3_ react at high temperatures to form yttrium aluminum garnet Y_3_Al_5_O_12_ (PDF# 33-0040). Owing to its high melting point and low oxygen diffusivity, Y_3_Al_5_O_12_ not only promotes densification by retarding grain boundary migration but also optimizes the oriented growth of β-Si_3_N_4_ grains by controlling the viscosity of the liquid phase.

**Figure 9:**
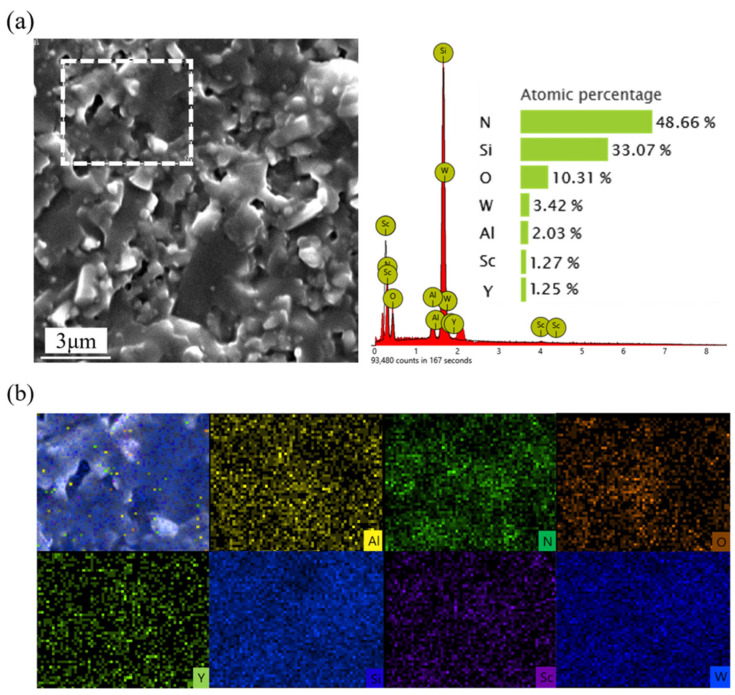
SEM micrographs of the fracture surface of SNS3: (**a**) EDS spectrum from mapping analysis; (**b**) elemental distribution maps.

Figure 9 presents the EDS energy spectrum analysis and element distribution maps of the fracture surface of the tool material SNS3. The presence and distribution of the Sc, W, and O elements confirm the independent formation and uniform dispersion of the Sc_2_W_3_O_12_ phase. Quantitative analysis reveals that the atomic percentages in the Sc-W-O enrichment region are approximately consistent with the theoretical stoichiometric ratio of Sc_2_W_3_O_12_, thereby further confirming the complete reaction between Sc_2_O_3_ and WO_3_, as well as the chemical stability of the target phase Sc_2_W_3_O_12_. Additionally, the distribution of major elements such as Si, N, Y, and Al demonstrates sufficient elemental diffusion during the SPS sintering process.

### 3.5. Toughening and Strengthening Mechanisms

In multiphase composite ceramic systems, the disparity in thermal expansion behavior between the reinforcing phase and the matrix phase can result in the development of a distinct internal stress field distribution at the phase interfaces. This stress regulation mechanism is an important approach for strengthening and toughening composite ceramic materials. According to the formula derived by Ohji [38], the calculation formula for the residual stress in composite ceramic materials is as follows:

(3) $$P_{\mathrm{res}}=\frac{2\Delta \alpha \Delta TE_{m}}{\left(1+v_{m})+2\beta (1-2v_{p}\right)}$$

(4) $$\sigma =P_{\mathrm{res}}{\left(\frac{r_{p}}{R}\right)}^{3}$$

(5) $$\sigma_{t}=-\frac{1}{2}P_{\mathrm{res}}{\left(\frac{r_{p}}{R}\right)}^{3}$$

where Δ*α* = *α*_p_ − *α*_m_ and *β* = *E*_m_ − *E*_p_, the subscripts m and p represent the matrix and the particles, respectively, *E* and *v* denote the elastic modulus and Poisson’s ratio, Δ*T* is the temperature difference between the sintering temperature *T*_p_ and the ambient room temperature *T*_r_ as the sintering temperature cools to the ambient temperature, *σ*_r_ and *σ*_t_ are the radial and tangential stresses within the matrix, *R* is the distance from a point in the stress field to the particle center, and *R*_p_ is the particle radius.

From the aforementioned calculation formula, it can be observed that, during the sintering and cooling process of the cutting tool material, on the one hand, the matrix is compressed due to the expansion effect of the negative thermal expansion phase, which offsets a portion of the residual tensile stress, thereby reducing the tensile stress level within the matrix. On the other hand, if the compressive stress induced by the expansion effect of the negative thermal expansion phase exceeds the original residual tensile stress within the matrix, the cutting tool material, as a whole, will exhibit a compressive stress state.

**Figure 10:**
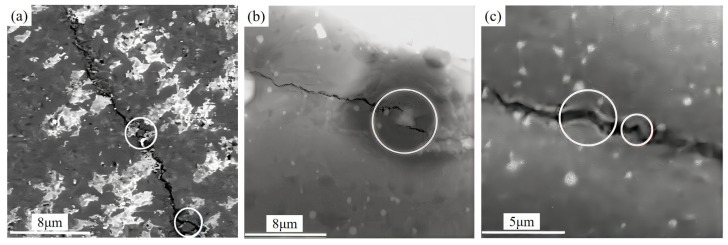
Crack propagation morphology on a polished SNS3 surface. (**a**) Crack branching, (**b**) crack bridging, and (**c**) crack deflection.

**Figure 11:**
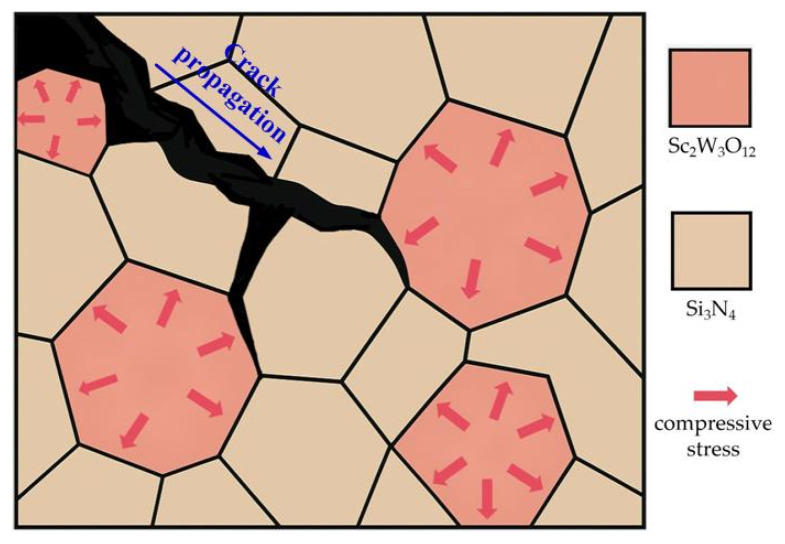
Compressive stress field formed at the Si_3_N_4_/Sc_2_W_3_O_12_ grain boundaries.

The crack propagation characteristics on the polished surface of the composite ceramic cutting tool material SNS3 are illustrated in Figure 10. The observed phenomena, including crack bifurcation (Figure 10a), crack bridging (Figure 10b), and crack deflection (Figure 10c), correspond to typical fracture mechanics behavior [39,40]. In the Si_3_N_4_/Sc_2_W_3_O_12_ composite ceramic cutting tool material system, the negative thermal expansion (NTE) characteristic of Sc_2_W_3_O_12_ plays a critical role in material toughening by constructing a residual compressive stress field through the thermal expansion accommodation. During the sintering cooling process, the negative thermal expansion behavior of Sc_2_W_3_O_12_ and the shrinkage of the Si_3_N_4_ matrix generate opposing deformation constraints, leading to a gradient distribution of compressive stress fields at the grain boundaries (as shown in Figure 11). The inhibitory effect of the compressive stress field on crack propagation is manifested in two aspects. Firstly, the compression of the grain boundary region inhibits the initiation of microcracks and delays the propagation of the main crack. Secondly, a closure stress component forms at the crack tip, forcing the crack path to deflect, branch, or bridge. These mechanisms require the cracks to overcome energy dissipation associated with the tortuous path and the compressive stress barrier, ultimately enhancing the material’s fracture toughness.

**Figure 12:**
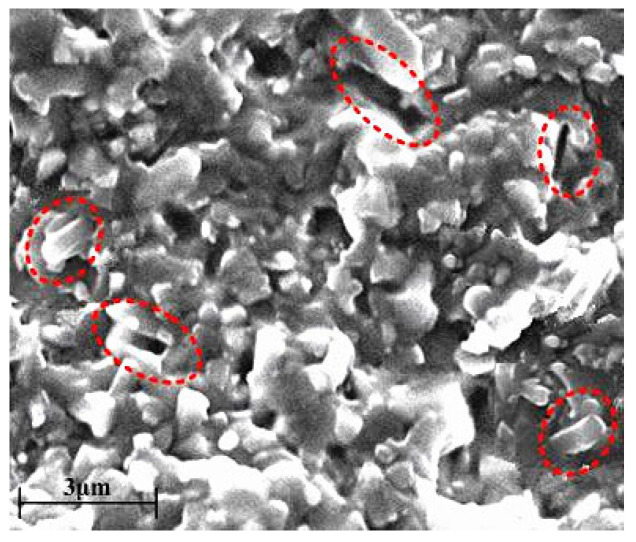
Pull-out of β-Si_3_N_4_ columnar grains.

Furthermore, the synergistic effect of the microstructural characteristics and the stress field significantly enhanced the material’s toughening mechanism. As shown in Figure 6d, Sc_2_W_3_O_12_ forms a uniform, spherical, chain-like network structure along the β-Si_3_N_4_ grain boundaries, effectively inhibiting crack propagation and promoting crack branching, bridging, and deflection. As illustrated in Figure 12, the pull-out of β-Si3N4 columnar crystals and the resultant voids (indicated by the red elliptical dotted box) occur due to the interfacial shear stress between β-Si_3_N_4_ columnar crystals and the surrounding Sc_2_W_3_O_12_, reaching the shear yield strength as cracks propagate. Owing to the elevated stiffness and strong tensile capability of the columnar grains, they are pulled out from the spherical, chain-like network structure of Sc_2_W_3_O_12_. The pulled out β-Si_3_N_4_ grains exhibit an estimated length of ~1.5–2.0 μm and diameter of ~0.2–0.5 μm, corresponding to a length-to-diameter ratio of approximately 3:10. This aspect ratio is generally considered favorable for promoting crack bridging and pull-out toughening. Such columnar grains can effectively hinder crack propagation by bridging crack faces and dissipating energy during the pull-out process, thereby contributing to the improvement in fracture toughness.

Meanwhile, the compressive stress field generated by thermal expansion fitting promotes the crack propagation preferentially along the Sc-W-O/Si_3_N_4_ matrix interface rather than through the transgranular fracture. This interface-dominated fracture mode not only increases the fracture surface area but also significantly enhances the energy dissipation efficiency. Additionally, the spherical, chain-like network structure of Sc_2_W_3_O_12_ facilitates stress transfer and transforms localized compressive stress into a macroscopic gradient field. Consequently, during crack propagation, it ensures that the crack remains continuously subjected to closing stress, thereby surpassing the inherent toughness limit in traditional dispersion-strengthened materials.

### 3.6. Cutting Performance

The orthogonal test results of single-factor dry cutting of AISI 1045 steel using the SNS3 cutting tool are presented in Table 5. A comprehensive analysis of the cutting temperature, cutting force, and workpiece surface quality indicates that the optimal cutting performance of the SNS3 tool is achieved at cutting parameters of *v_c_* = 150 m/min, *a_p_* = 0.1, and *f* = 0.101 mm/rev. Under the same cutting parameters, a comparative analysis of the cutting performance between the SNS3 and SNS0 tools was conducted, with the results presented in Table 6.

As presented in Table 6, the low cutting force and low-temperature characteristics of the SNS0 tool can be attributed to its inferior mechanical properties and inadequate thermal conductivity. Despite a significant amount of heat being dissipated through the chips, the presence of numerous pores and microcracks in the SNS0 material weakens the grain boundaries, thereby reducing the tool’s thermal shock resistance and accelerating its failure. In contrast, the SNS3 tool achieves simultaneous optimization of interface stress buffering and grain boundary heat flow guidance via the spherical chain network structure of Sc_2_W_3_O_12_ at the grain boundaries. The distribution of Sc_2_W_3_O_12_ facilitates enhanced heat dissipation along the grain boundaries, while the strong interface bonding force ensures that the cutting energy is more uniformly distributed throughout the tool body. Consequently, a stable temperature rise at the tool–chip interface is achieved. Although the measured temperature of the SNS3 tool exceeds that of the SNS0 tool, it actively regulates the thermodynamic equilibrium through uniform thermal shock resistance. The strong interface bonding enables the cutting energy to be dissipated via controlled heat conduction and in the form of chips, which reflects its superior load bearing capacity and significantly delays the onset of thermal fatigue failure.

**Figure 13:**
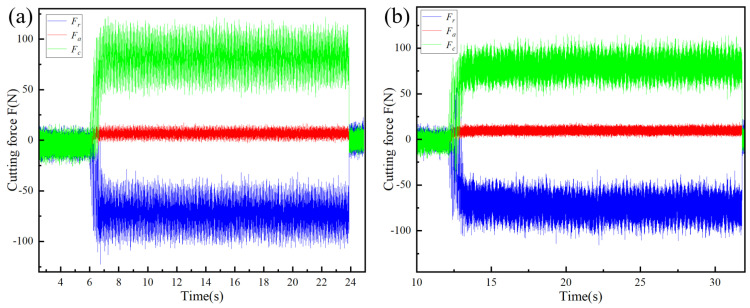
Cutting force evolution over time during machining with composite ceramic tools: (**a**) SNS0 and (**b**) SNS3.

From the dynamic response characteristics of the cutting force illustrated in Figure 13, it is evident that the cutting performance of SNS3 is substantially superior to that of SNS0. The main cutting force (*F*_c_) and radial force (*F*_r_) of SNS0 exhibit irregular and intense fluctuations, with occasional zero-value drifts observed at specific time points (e.g., at 8 s and 15 s), which signify significant instability in the dynamic interaction between the tool and the workpiece. In contrast, the main cutting force curve of SNS3 exhibits a narrower fluctuation range, with the oscillation amplitude of the radial force significantly reduced (amplitude < 20 N), and tends toward a smooth transition. Furthermore, the dynamic disturbances during the latter phase of the cutting process exhibit systematic attenuation, indicating that the gradient-optimized stress field of SNS3 tool material can effectively regulate the dissipation of the cutting energy.

**Figure 14:**
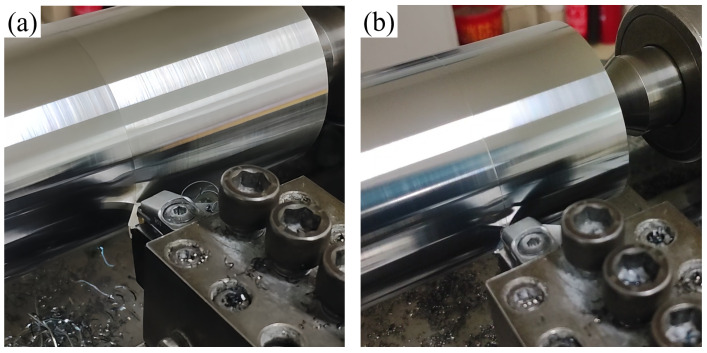
Workpiece surface morphology machined by different tools: (**a**) SNS0 and (**b**) SNS3.

Figure 14 presents the surface morphology of workpieces machined using the SNS0 and SNS3 cutting tools. The surface of the workpiece machined with the SNS0 tool exhibits pronounced periodic chatter marks and circular tool scratches (as shown in Figure 14a), which can originate from edge chipping caused by intergranular tensile stress, subsequently triggering periodic high-frequency vibrations. In contrast, the surface machined with the SNS3 tool exhibits a near-mirror finish of high quality (as shown in Figure 14b), highlighting the optimized geometric accuracy and superior optical reflectivity. These morphological differences are closely associated with the microstructural and thermophysical characteristics of the cutting tools. Specifically, SNS3 establishes a gradient compressive stress field via the negative thermal expansion effect of Sc_2_W_3_O_12_, effectively inhibiting crack propagation and improving fracture toughness. Its grain boundary strengthening mechanism effectively reduces the likelihood of dynamic edge instability. Furthermore, the thermal shock resistance of the negative thermal expansion of Sc_2_W_3_O_12_ ensures geometric stability under cutting-induced thermal loads, enabling SNS3 to maintain uniform cutting contact during high-speed interrupted cutting. In addition, the uniform stress field distribution and enhanced grain boundary bonding force of SNS3 contribute to superior surface accuracy during machining.

**Figure 15:**
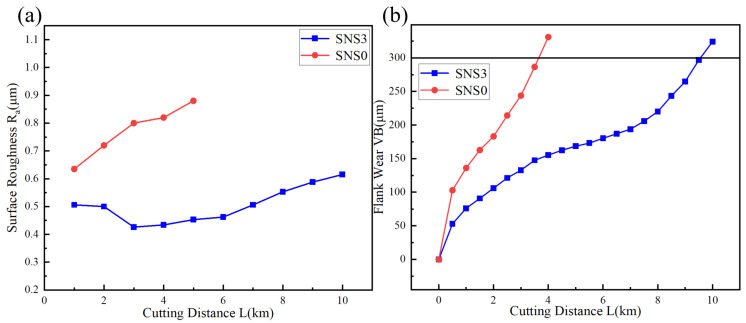
Evolution of the (**a**) workpiece surface roughness (Ra) and (**b**) flank wear (VB) with cutting distance (L).

Figure 15a illustrates the variation in surface roughness (Ra) of the workpiece with cutting distance (L) during the machining process using the two types of tools. Among them, the surface roughness produced by the SNS0 tool shows a continuously increasing trend, whereas the surface roughness generated by the SNS3 tool remains within a narrow low value range throughout the entire cutting process, particularly demonstrating near-horizontal stability in the middle and later stages. This behavior can be attributed to the low cutting force, which enhances the uniformity of plastic deformation on the workpiece’s surface layer, as well as the low temperature rise, which effectively suppresses thermal dimensional drift and oxidation-induced adhesion. Meanwhile, the SNS3 composite ceramic cutting tool, which exhibits the negative thermal expansion behavior of Sc_2_W_3_O_12_, possesses outstanding mechanical properties and superior wear resistance. These attributes ensure the long-term stability of the cutting edge geometry. Figure 15b presents the evolution of flank wear (VB) with cutting distance (L). The wear curve of the SNS0 tool exhibits an approximately linear increase, with an effective cutting distance of only about 3500 m. In contrast, the SNS3 tool demonstrates a typical three-stage wear behavior, achieving an effective cutting distance of up to 9500 m. A combined analysis of Figure 13a,b indicates that the linear wear behavior of the SNS0 tool directly corresponds to the continuous deterioration of the workpiece surface roughness (Ra). In contrast, the SNS3 tool exhibits a three-stage wear progression that enables controlled variation of the Ra, ensuring better surface quality throughout the cutting process.

**Figure 16:**
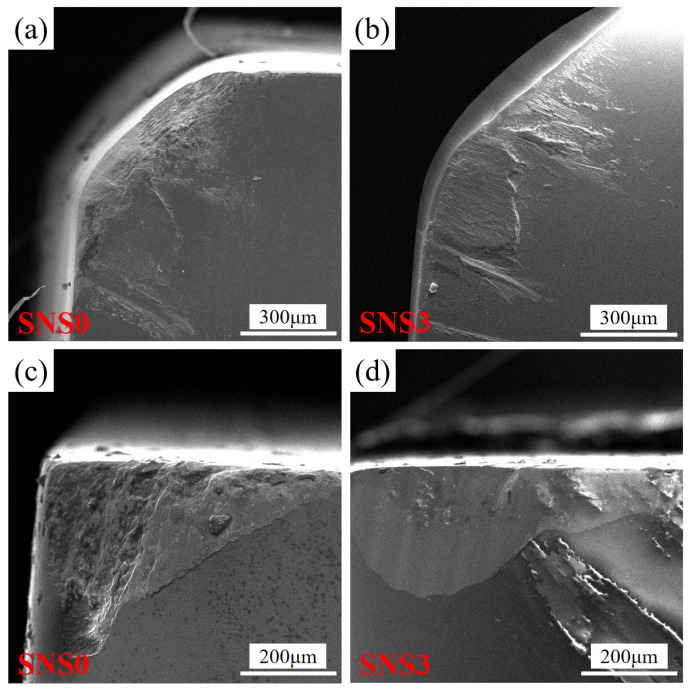
SEM of the wear morphology of the SNS0 and SNS3 composite ceramic tools: (**a**) rake face of SNS0, (**b**) rake face of SNS3, (**c**) flank face of SNS0, and (**d**) flank face of SNS3.

**Figure 17:**
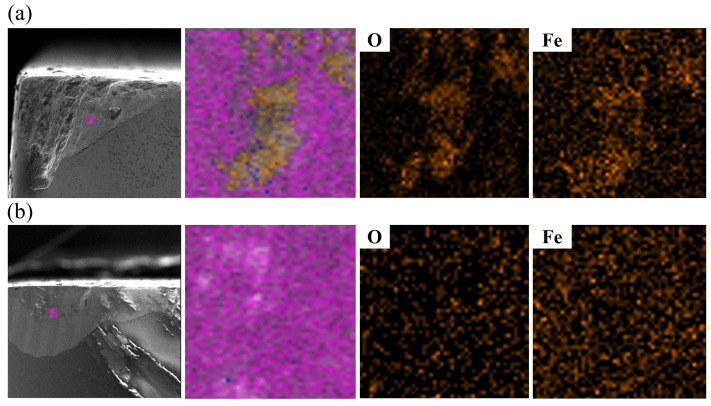
SEM and EDS of the flank face wear of the (**a**) SNS0 and (**b**) SNS3 composite ceramic tools.

As shown in Figure 14 and Figure 15, both the rake faces of the SNS0 and SNS3 tools experienced boundary groove wear and adhesive wear, while the flank faces underwent boundary groove wear, abrasive wear, adhesive wear, and oxidative wear. As shown in Figure 16a,b, distinct groove wear can be observed on both the chip outflow direction of the rake face and the flank face of the SNS0 and SNS3 tools. Under the high-temperature and high-pressure conditions of cutting, the chips and workpiece material continuously move along the rake and flank faces of the tool, causing adhesion between the tool and workpiece materials at the cutting interface. Under the combined effects of intense friction-induced high stress and elevated temperature between the tool and the chip, microscopic defects in the tool material cause surface fracture, resulting in the detachment of material by the chip and the formation of irregular pits on both the rake and flank faces, thereby leading to adhesive wear. In Figure 17, the energy spectrum reveals that a large amount of Fe element, the main component of the workpiece material, was detected. Under the combined effect of the continuous friction between burrs on the chip impacting the cutting edge and burrs on the workpiece surface acting on the flank face, the boundary stress and temperature gradients at the tool–workpiece contact zone become elevated, leading to stress concentration and the formation of localized thermal stress cracks, which ultimately result in boundary groove wear on the flank face (as shown in Figure 16c,d). Meanwhile, the Fe element from the workpiece material was diffused into the grooves, causing adhesion of the workpiece material at the groove sites. This not only enhances the affinity of the workpiece material but also alters the direction of the stress impact, accelerating the propagation of thermal cracks. When adhesion reaches a certain extent, the bonded material detaches under the impact force, carrying away portions of the tool material, which results in tool wear until the flank wear reaches the bluntness criterion.

Additionally, a significant amount of the O element was detected in the EDS spectra of Figure 17, indicating that oxidative wear occurred on the flank faces of both the SNS0 and SNS3 tools. This indicates that the temperature in the contact area between the flank face and the chip was sufficiently high to reach the oxidation temperature of the tool. Since the matrix of SNS3 tools are silicon nitride, which has relatively poor thermal conductivity, when adhesion occurs between the tool, chip, and workpiece, the cutting heat is not promptly removed by the chip but remains on the tool. This causes elevated temperatures in the tool–chip contact zone, leading to tool oxidation, which reduces the tool’s strength and accelerates wear. Moreover, due to the lower hardness of the SNS0 tool compared to the SNS3 tool, severe scratching occurs on the flank face of the SNS0 tool during cutting, resulting in more pronounced abrasive wear. Meanwhile, the distribution of the spherical, chain-like Sc_2_W_3_O_12_ structure in SNS3 effectively hinders abrasive indentation and crack propagation. As a result, the compressive stress gradient induced by the negative thermal expansion of Sc_2_W_3_O_12_ significantly suppresses microstructural damage caused by thermal shock and mechanical vibrations, thereby substantially reducing the wear rate of the SNS3 tool. As shown in Figure 16, the rake and flank faces of the SNS0 tool exhibit more severe wear compared to those of the SNS3 tool. This is primarily attributed to the effect of Sc_2_W_3_O_12_, which alleviates the residual tensile stress within the matrix through its negative thermal expansion behavior. The resulting gradient compressive stress field enhances the tool’s thermomechanical load bearing capacity and reinforces the grain boundaries to inhibit crack propagation—consistent with the steady and gradual extension observed in the stable wear stage of the SNS3 tool in the wear curve shown in Figure 15b. The primary failure modes of the SNS0 and SNS3 tools are brittle spalling and damage on both the rake and flank faces.

## 4. Conclusions

In this study, Si_3_N_4_-based composite ceramic cutting tools reinforced with the negative thermal expansion phase of Sc_2_W_3_O_12_ were fabricated via in situ synthesis combined with spark plasma sintering (SPS). The composites’ thermal expansion coefficient, residual stress, mechanical properties, microstructure, toughening mechanisms, and cutting performance were systematically investigated.

The addition of Sc_2_W_3_O_12_ effectively reduced the thermal expansion coefficient and regulated the internal residual stresses, enhancing the grain boundary bonding strength and inhibiting crack propagation. Under optimized sintering conditions (1650 °C, 10 min holding time, 40 MPa pressure), the composite containing 12 wt.% Sc_2_W_3_O_12_ exhibited superior mechanical properties, including a flexural strength of 712.4 ± 30 MPa, fracture toughness of 7.5 ± 0.3 MPa·m^1/2^, hardness of 16.3 ± 0.3 GPa, relative density of 98.8 ± 0.2%, and a reduced thermal expansion coefficient of 2.81 × 10^−6^·K^−1^. The residual tensile stress decreased to 66.5 ± 8.7 MPa, while the compressive stress increased to 57.9 ± 4.5 MPa.

The negative thermal expansion behavior of Sc_2_W_3_O_12_ contributed to reducing residual tensile stresses and enhancing compressive stresses, further improving the composite’s flexural strength. The spherical, chain-like network structure of Sc_2_W_3_O_12_ distributed along the β-Si_3_N_4_ grain boundaries effectively suppressed crack propagation through mechanisms such as branching, bridging, and deflection. The pull-out of β-Si_3_N_4_ columnar grains and associated voids dissipated the fracture energy, enhancing the toughness.

Finally, the incorporation of Sc_2_W_3_O_12_ improved the surface quality and cutting reliability of the tools. Under dry cutting of 45# steel, the primary failure modes were brittle spalling and chipping on the rake and flank faces, accompanied by boundary groove wear, adhesive wear, abrasive wear, and oxidative wear.

Future research should focus on further enhancing the mechanical and cutting performance of Si_3_N_4_/Sc_2_W_3_O_12_ composite ceramic tools by incorporating additional hard phases such as WC, TiC, or (W,Ti)C to improve the hardness and flexural strength. Optimization of the sintering parameters, including varied heating rates and staged holding and pressure, may improve material densification and high-temperature mechanical and thermal shock resistance. Moreover, advanced multiscale computational modeling combined with machine learning techniques could be employed to explore the dynamic evolution of composition–structure–property relationships, facilitating the intelligent design and optimization of composite ceramic tool materials.

## Figures and Tables

**Table 1:** Basic physical properties of the materials employed in this study.

Raw Material	Particle Size (μm)	Purity	Thermal Expansion Coefficient (α/10^−6^·K^−1^)	Elastic Modulus (E/GPa)	Poisson’s Ratio
α-Si_3_N_4_	0.3	99.99%	3.0	300	0.26
WO_3_	0.5	99.9%	7.0	110	0.26
Sc_2_O_3_	0.5	99.9%	8.5	140	0.24
α-Al_2_O_3_	0.3	99.99%	7.2	350	0.23
Y_2_O_3_	0.5	99.9%	8.0	150	0.29

**Table 2:** Compositions (wt.%) of various composite samples.

Composites	Sc_2_W_3_O_12_	WO_3_	Sc_2_O_3_
SNS0	0.00	0.00	0.00
SNS1	8.00	6.68	1.32
SNS2	10.00	8.35	1.65
SNS3	12.00	10.02	1.98
SNS4	16.00	13.36	2.64

**Table 3:** Composition ratios (wt.%).

Code	Si_3_N_4_/wt.%	Al_2_O_3_/wt.%	Y_2_O_3_/wt.%	WO_3_/wt.%	Sc_2_O_3_/wt.%
SNS0	90.00	3.00	7.00	0.00	0.00
SNS1	82.00	3.00	7.00	6.68	1.32
SNS2	80.00	3.00	7.00	8.35	1.65
SNS3	78.00	3.00	7.00	10.02	1.98
SNS4	74.00	3.00	7.00	13.36	2.64

**Table 4:** Tool geometry parameters.

Clearance Angle *α*°	Rake Angle *γ*°	Edge Inclination Angle	Cutting Edge Angle	Corner Radius *γ*_ε_	Chamfer
5°	−5°	0°	45°	0.5 mm	−10° × 0.1 mm

**Table 5:** Orthogonal test results of single-factor dry cutting of AISI 1045 steel using the SNS3 tool.

No.	Cutting Speed *v_c_* (m/min)	Cutting Depth *a_p_* (mm)	Axial Force *F_a_* (N)	Radial *F_r_* (N)	Main Cutting Force *F_c_* (N)	Cutting Temperature T (°C)	Surface Roughness *Ra* (μm)
1	100	0.05	23	69	81	79.5	1.24
2	100	0.1	22	70	90	141.3	1.202
3	100	0.15	28	84	103	163.7	1.324
4	100	0.2	37	91	114	221.6	1.389
5	150	0.05	23	79	92	128.4	0.499
6	150	0.1	21	80	89	205.7	0.452
7	150	0.15	26	96	97	228.9	0.572
8	150	0.2	35	101	131	314.1	0.637
9	200	0.05	24	73	97	75.9	0.679
10	200	0.1	21	102	119	134.8	0.632
11	200	0.15	31	121	146	161.1	0.752
12	200	0.2	43	167	179	247.3	0.817
13	250	0.05	26	76	102	158.6	0.425
14	250	0.1	25	81	108	217.4	0.398
15	250	0.15	31	149	156	246.9	0.498
16	250	0.2	48	186	201	324.2	0.563

**Table 6:** Cutting force and cutting temperature.

Type	Axial Force *F_a_* (N)	Radial Force *F_r_* (N)	Main Cutting Force *F_c_* (N)	Cutting Temperature T (°C)
SNS0	18	68	82	139.1
SNS3	19	76	83	176

## Data Availability

The original contributions presented in this study are included in the article. Further inquiries can be directed to the corresponding author.
